# Well-differentiated gall bladder hepatoid carcinoma producing alpha-fetoprotein: a case report

**DOI:** 10.4076/1752-1947-3-7303

**Published:** 2009-06-26

**Authors:** Ching-Yun Kao, Chian-Ro Chang, Hung Chiang, How-Tshung Chen, Ma Shih-Ming, Che-Yu Cheng

**Affiliations:** 1 West Garden Hospital, Taipei, Taiwan, ROC; 2 Taipei Institute of Pathology, Taipei, Taiwan, ROC

## Abstract

**Introduction:**

Gall bladder carcinoma is rare, and metastatic gall bladder carcinoma from hepatocellular carcinoma has been reported in only a few patients.

**Case presentation:**

We present a 73-year-old man with a history of hepatitis B virus-related liver cirrhosis and hepatocellular carcinoma. He received transcatheter arterial chemoembolization, and was diagnosed to have an alpha-fetoprotein producing gall bladder tumor with intraluminal growth. Open cholecystectomy was performed. Pathologic examination of the lesion revealed a well-differentiated hepatoid carcinoma. The lesion was thought most likely to be a metastatic lesion from previous hepatocellular carcinoma. His alpha-fetoprotein level dropped to normal levels five months after the surgery.

**Conclusion:**

This unusual intraluminal growing tumor proved to be a well-differentiated hepatoid carcinoma, most likely a metastatic lesion from previous hepatocellular carcinoma. This case reminds clinicians that in looking for likely hepatocellular carcinoma recurrence, when no detectable hepatic lesion can account for an elevated alpha-fetoprotein level, the gall bladder should be included in the search for the site of metastasis.

## Introduction

Gall bladder carcinoma is a rare disease, whether primary or metastatic. It is usually diagnosed at a late stage due to asymptomatic properties when small. Sometimes carcinoma from the gall bladder also produces alpha-fetoprotein (α-FP) because the gall bladder shares the same embryologic origin as the liver. Only a handful of cases of α-FP producing gall bladder carcinoma had been reported in the literature, and many of them have been from Japan. Cell types of these reported cases include clear cell carcinoma, pleomorphic carcinoma or undifferentiated carcinoma. Here we present a rare case of a well-differentiated gall bladder hepatoid carcinoma producing α-FP after treatment of primary hepatocellular carcinoma (HCC).

## Case presentation

A 73-year-old man visited our emergency room (ER) in October 2004 with right upper abdominal pain and fever for the previous two days. Vital signs on arrival at the ER were blood pressure (BP): 161/107 mmHg, pulse rate (PR): 90/minute, respiratory rate (RR): 20/minute, and body temperature (BT): 38.9ºC. His consciousness was unaffected but he had an acutely ill appearance. Tracing back his medical history, he had hepatitis B virus (HBV)-related liver cirrhosis (Child-Pugh Classification Grade A), and in August 1999, a small HCC (Segment 5 of the liver, diameter less than 2 cm) was diagnosed. He received transcatheter arterial chemoembolization (TACE) at a medical center and a second TACE again in May 2001 for recurrent HCC. He declined further TACE for residual recurrent lesions. Therefore oral chemotherapy with the regimen tegafur/uracil (100 mg/224 mg) had been prescribed to him from December 2001 to November 2003, and was ceased due to loss at follow-up.

**Figure 1:**
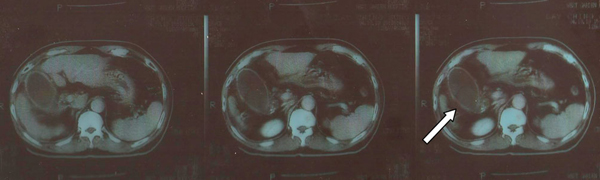
**Computed tomography findings**. Gall stones, diffusely thickened gall bladder wall with increased contrast enhancement, and a hypodense mass lesion (arrow) without contrast enhancement are noted in the gall bladder lumen.

Physical examination in the ER found no yellowish skin, no icteric sclera but moderate right upper quadrant (RUQ) abdominal pain. Murphy's sign was positive. Bowel sounds were normal and his abdomen was soft, with no rebounding pain. There was no shifting dullness. Lab data included: white blood cell count (WBC): 14,520/mm^3^ (Neu/Lym: 83.5/6.4%), platelets: 169 k/mm^3^, total and direct (T/D) bilirubin: 1.4/0.6 mg/dL, serum glutamic-oxaloacetic transaminase/serum glutamic-pyruvic transaminase (sGOT/sGPT): 38/32 U/L, albumin (Alb): 3.9 gm/dL, C-reactive protein: 11.9 mg/dL, NH_3_: 98 mcg/dL, and α-FP level: 231.1 ng/mL. Abdominal echogram revealed a thickened gall bladder wall, gall stones and a suspected tumor mass in the distended gall bladder. An abdominal computed tomography (CT) scan showed liver cirrhosis, gall stones, diffusely thickened gall bladder wall with increased contrast enhancement compatible with acute cholecystitis, and a hypodense mass lesion without contrast enhancement in the gall bladder (Figure 1). There was no roentgenographic evidence of a suspected recurrent lesion in the liver parenchyma at that time.

**Figure 2:**
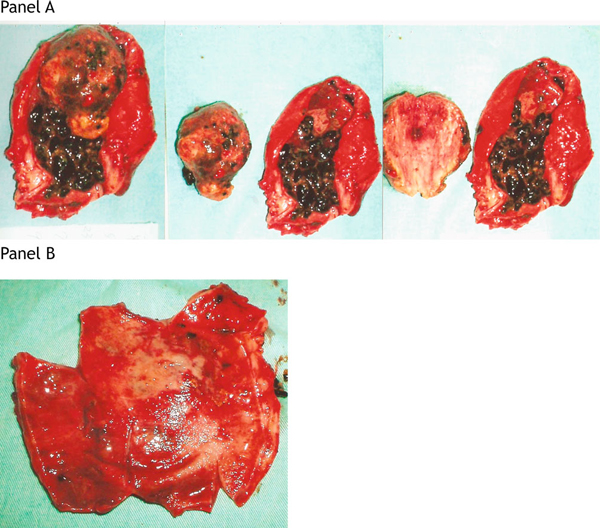
**Operative findings**. **Panel A.** Inflammatory gall bladder contains multiple gall stones and a tumor measuring 6.0 × 4.0 × 2.0 cm. **Panel B.** The mucosal surface of the gall bladder is hemorrhagic but smooth without evident tumorous or ulcerative lesions.

An open cholecystectomy via a right subcostal incision was performed on the next day. A distended gall bladder with multiple pigmented gall stones and a tumor mass, 6.0 × 4.0 × 2.0 cm in size, were found in the gall bladder lumen. When seen, the mass had already detached from the mucosa at the opening of the sac and no stalk could be identified (Figure 2, Panel A). The mucosal surface of the gall bladder wall was smooth without evident tumorous or ulcerative lesions (Figure 2, Panel B).

**Figure 3:**
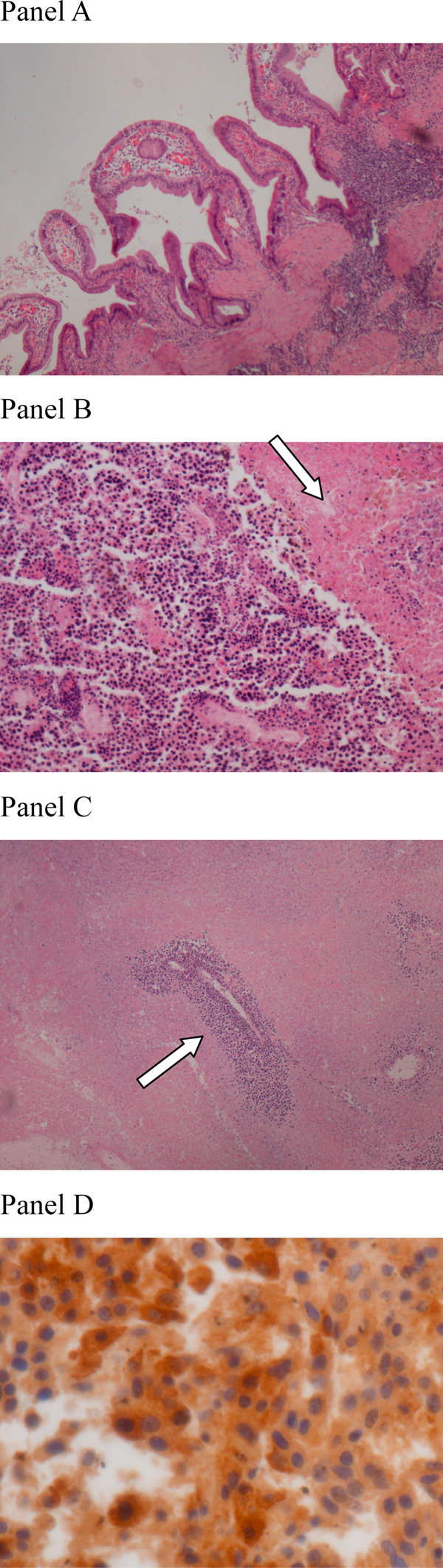
**Pathologic examination**. **Panel A.** Gall bladder: Neutrophil and lymphocyte infiltration indicate acute and chronic inflammation compatible with cholecystitis. **Panel B.** Hepatoid carcinoma with the histologic feature of uniform cells with eosinophilic cytoplasm arranged in a tile-like array or microtrabecular pattern. Infarction necrosis is seen in the right upper field (arrow). **Panel C.** Viable tumor cells are mostly found surrounding the blood vessels (arrow). **Panel D.** Immunostaining for alpha-fetoprotein demonstrates varied staining intensity in the cytoplasm of the tumor cells.

Microscopic pathologic examination of the gall bladder revealed heavy neutrophil and lymphocyte infiltration as in ordinary acute and chronic inflammation. The mucosa was congested and showed no evidence of tumor invasion. The vessels in the gall bladder wall were free from tumor emboli (Figure 3, Panel A).

The sections of the tumor showed extensive infarction necrosis. The viable tumor cells were seen as uniform polygonal cells with eosinophilic granular cytoplasm arranged in tiled array or microtrabecular pattern mostly surrounding the blood vessels (Figure 3, Panel B and C). Immunohistochemistry staining with α-FP antibody demonstrated varied positive staining intensity in the cytoplasm of the tumor cells (Figure 3, Panel D). Other antibodies including CK7, chromogranin, and synaptophysin were all negative, excluding the possibility of carcinoid tumor or adenocarcinoma.

The patient recovered from the surgery well without major or minor complications and was discharged one week after the surgery. He received regular follow-up in our gastrointestinal (GI) outpatient department and his α-FP levels went down to 3.1 ng/mL in March 2005, and to 3.08 ng/mL in August 2005, five and 10 months after cholecystectomy, respectively. He died in July 2006 in another hospital due to upper gastrointestinal bleeding from a hemorrhagic duodenal ulcer complicated by hepatorenal syndrome.

## Discussion

α-FP is a serum glycoprotein elevating in pregnancy and pathologic tumor growth such as HCC and germ cell tumors. It is used to evaluate treatment and to detect recurrence of these tumors. Sometimes, gall bladder tumors also produce α-FP because the gall bladder and liver have identical embryologic origin. Recurrence or metastasis of these tumors can be detected by a relapsing α-FP level [1].

Gall bladder tumors, either primary or metastasis, are rare and often diagnosed at a late stage because of asymptomatic properties when small and without biliary tract obstruction. Primary α-FP producing gall bladder tumors consist of several cell types, including undifferentiated carcinoma, papillary clear cell carcinoma and hepatoid carcinoma. It is possible that these α-FP producing gall bladder tumors may have cell types within the two extremes of the spectrum, undifferentiated carcinoma and well differentiated hepatoid carcinoma [2]. We regard our patient to be a unique rare case of α-FP producing well-differentiated hepatoid carcinoma presenting as an intraluminal growing tumor mass in the gall bladder sac without wall invasion. No identical scenario has been reported in the literature.

Undifferentiated gall gladder carcinoma has been reported in an autopsy specimen by Ng and Ng [3]. Brown and Roberts described a papillary type gall bladder adenocarcinoma with α-FP producing properties [4]. St Laurent *et al.* also reported two α-FP producing gall bladder tumors with direct invasion to liver showing cell types of primary undifferentiated carcinoma and a poorly differentiated tumor with a wide range of polymorphism containing hepatoid differentiation, respectively [4]. As for metastatic lesions, gall bladder carcinomas, although rare, may metastasize to the liver by direct invasion [2] and with distant metastasis to the lung [1]. HCC metastasis to the gall bladder is much rarer. Terasaki *et al.* reported an autopsy result showing HCC with gall bladder metastasis presenting with massive intraluminal growth [5]. Chiba *et al.* reported another autopsy case of HCC with gall bladder metastasis also presenting with intraluminal tumor growth [6]. Both of these two autopsied HCC metastases to the gall bladder were continuous with the gall bladder wall and tumor emboli in dilated vessel lumens, and the latter character led to the postulation of vessel route metastasis. A lesser possibility of the mechanism of direct invasion was discussed in the literature because it is quite rare for HCC to destroy the muscle layer and collagen fibers of the gall bladder wall [6].

In our patient, an intraluminal growing gall bladder tumor with intact gall bladder wall was found and microscopic examination revealed uniformly well differentiated hepatoid carcinoma without tumor emboli in vessels. The patient recovered from the operation without major or minor complications and a normal α-FP level was noted 10 months after the surgery, and 20 months after cessation of palliative oral chemotherapy. We believe that primary α-FP producing gall bladder tumors have undifferentiated cell type or polymorphic pattern, and that the α-FP production may be the result of dedifferentiation. Since our case appeared to be a uniformly well differentiated hepatoid carcinoma after treatment of HCC, it is most likely a metastatic lesion. Arakawa *et al.* reported ectopic liver tissue with hepatocarcinogenesis [7] which is less likely in our patient since there were no normal hepatocytes present in the gall bladder tumor.

## Conclusion

In summary, this unusual intraluminal growing tumor proved to be a well-differentiated hepatoid carcinoma and is most likely a metastatic lesion from previous HCC. This case reminds clinicians that, in looking for likely HCC recurrence, when no detectable hepatic lesion can account for elevated α-FP levels, the gall bladder should be included in the search for the site of metastasis.

## Abbreviations

Alb: albumin; BP: blood pressure; BT: body temperature; CT: computed tomography; ER: emergency room; HBV: hepatitis B virus; HCC: hepatocellular carcinoma; PR: pulse rate; RR: respiratory rate; RUQ: right upper quadrant; sGOT/sGPT: serum glutamic-oxaloacetic transaminase/serum glutamic-pyruvic transaminase; TACE: transcatheter arterial chemoembolization; T/D: total and direct; α-FP: alpha-fetoprotein.

## Consent

Written informed consent was obtained from the patient's family for publication of this case report and any accompanying images. A copy of the written consent is available for review by the Editor-in-Chief of this journal.

## Competing interests

The authors declare that they have no competing interests.

## Authors' contributions

CY was the major contributor in writing the manuscript. CR performed the operation (open cholecystectomy) and initiated the idea for this case report. CH performed the histologic examination as well as the special staining (α-FP stain) of the specimen. HT treated and followed-up this patient. SM reviewed related articles and provided information on the nature and course of HCC and its setting in Taiwan. CY interpreted the image study (CT scan) and provided radiologic information about this case. All authors read and approved the final manuscript.
